# A randomized controlled trial of an mHealth application with nursing interaction to promote quality of life among community-dwelling older adults

**DOI:** 10.3389/fpsyt.2022.978416

**Published:** 2022-10-18

**Authors:** Arkers Kwan Ching Wong, Frances Kam Yuet Wong, Jonathan Bayuo, Karen Kit Sum Chow, Siu Man Wong, Athena Yin Lam Lee

**Affiliations:** ^1^School of Nursing, The Hong Kong Polytechnic University, Kowloon, Hong Kong SAR, China; ^2^The Hong Kong Lutheran Social Service, Ho Man Tin, Hong Kong SAR, China

**Keywords:** apps, health-social partnership, interactivity, mobile health, elderly, aged

## Abstract

**Significance:**

Using mHealth apps alone at home without the support of healthcare experts could mean that older adults might not fully utilize the functions of the apps, recognize their benefits, and sustain their use. Incorporating an integrated health-social partnership model to support the app usage when further help is needed by the older adults might maximize the apps' benefits in the long term.

**Objectives:**

This study aimed to examine the benefits of adding nursing interaction supported by a health-social partnership model in the use of mHealth, and the sustained beneficial effects on psychological outcomes, including quality of life, self-efficacy, and depression, among older adults after the completion of the program.

**Methods:**

A three-arm, randomized controlled trial design was adopted. Community-dwelling older adults with chronic pain, hypertension, or diabetes, were randomly assigned to either the mHealth, mHealth with interactivity, or control group. Subjects in both the mHealth and the mHealth with interactivity groups received the mHealth application. In addition, the mHealth with interactivity group received support from a nurse case manager, who was supported by a health-social partnership team. mHealth apps and services from a nurse case manager were not provided to the control group. The primary outcome measure was quality of life, and secondary outcomes were self-efficacy and depression. Data were collected at pre-intervention (T1), post-intervention (T2), and at 3 months post-intervention to measure the sustained effect of the program.

**Results:**

There were 74 mHealth+I, 71 mHealth, and 76 control group subjects enrolled in the program. No statistically significant between-group, within-group, and interaction effects between group and time in both physical component summary (PCS) and mental component summary (MCS) scores were found among the three groups. The mHealth group showed an improvement in PCS and depression scores from T1 to T2, sustained at T3; while the mHealth+I group demonstrated improved self-efficacy from T1 to T2, with a decrease at T3.

**Conclusion:**

Adding nurse-directed telephone calls may be of little to no benefit at all in the long term. Future studies may consider a longer intervention period to build and sustain quality of life and self-efficacy levels among community-dwelling older adults.

**Clinical trial registration:**

www.ClinicalTrials.gov, identifier: NCT03878212.

## Introduction

In the past few years, mHealth applications (mHealth apps) have become a popular channel for older adults to acquire health knowledge and monitor their health conditions (1, 2). Statistics showed that up to the year 2022, more than 350,000 mHealth apps were available in the market, many of them promoting chronic disease self-management among community-dwelling older adults (3). Although the features of these apps vary, in general, they include, but are not limited to, the monitoring of vital signs, health notifications, self-health assessments, multimedia educational materials, and medical appointment reminders (4, 5). Multiple systematic reviews have confirmed that the apps have beneficial effects for older adults, leading to improved blood pressure (6), glycemic control (7), quality of life (8), and treatment adherence (9), and to reductions in hospital admissions (10).

During the COVID-19 pandemic, mental illnesses and the demand for psychological services reached an all-time high among older adults. This has shifted the attention of researchers and healthcare professionals from developing mHealth apps focused only on physical health to those concerned with both the physical and psychological health of users (11, 12). For example, an mHealth app targeting older adults with chronic obstructive pulmonary disease was developed to monitor not only their vital signs, but also to measure the impact of disease on their moods and emotions (13). However, since the users did not receive support from healthcare professionals even though they reported borderline vital signs and unstable emotional states in the app, more than half of participants stopped using the app and reported no relief in depression and anxiety after the program (13). Another study adopted an mHealth app with the aim of maintaining the physical and psychological health of patients with cardiovascular diseases by providing health information and installing a chat function for cardiac patients to contact healthcare professionals when they had urgent enquires (14). Similar to the previous study, approximately half of the participants showed very low usage of the app, as there was no active support from healthcare professionals. The negative results found in blood pressure, activation, and depression levels among the participants in this study also indicated that the app itself was no replacement for the physical presence of healthcare professionals (14, 15). The evidence also suggests that using an mHealth app alone without interaction with healthcare experts may induce a feeling of loneliness, which may lead to depression, lower self-efficacy, and a higher risk of developing a mental disorder (16–18).

In order to improve the psychological health of older adults, previous studies suggested that it is critical to have support from interdisciplinary health and social care professionals including nurses, social workers, and general practitioners (19–21). Commonly appointed as case managers and health educators, nurses are able to provide individualized comprehensive health assessments and care, assist with self-care activities, and offer basic counseling, such as general guidance and interpersonal communication. Social workers can also connect older adults with important psychosocial and financial resources, and general practitioners can prescribe and monitor psychobiological treatment regimes. In terms of mHealth programs, having these health and social experts provide continuous psychological health monitoring, assist and motivate older adults to make daily use of the app, and meet their individual needs, can subsequently lead to better psychological health and quality of life. However, to the best of our understanding, there is no study evaluating the effects of an mHealth program with the support of a health-social partnership team on the psychological outcomes of community-dwelling older adults. The current manuscript will present the methodology, interventions, results of the program, discussion of the benefits of adding nursing interaction supported by a health-social partnership model in the use of mHealth, as well as the sustained beneficial effects on psychological outcomes among older adults after the completion of the program. The findings of the study will provide evidence to inform policy makers and health providers of the importance of human interaction in the use of technology.

## Methods

### Study design and settings

This was a single-blinded, three-armed randomized controlled trial. The data collectors were blinded, while the interventionists and the participants were not. The participants were recruited through five community centers run under the auspices of a non-governmental organization. The study was conducted based on the Declaration of Helsinki principles and was registered at ClinicalTrials.gov (NCT03878212).

### Subjects, recruitment strategy, and randomization

Our app targeted the three most common health problems among community-dwelling older adults, as identified in the latest survey: pain, hypertension, and diabetes mellitus (22). Older adults with at least one of these problems were recruited to the program if they (1) were aged 60 or above and (2) had a smartphone. Excluded were those who (1) had already been involved in other mHealth programs, (2) had been recently hospitalized with a known psychiatric problem within the last 6 months, (3) were bedbound, or (4) who did not have Internet coverage at home.

Simple random sampling was employed to select the participants since it only involves a single random selection and requires little advance knowledge about the participants. Eligible participants were approached in the community centers by a designated staff member. Those who agreed to participate were asked to sign a consent form and were assigned to one of the three groups in this study (i.e., mHealth+I, mHealth, and control) through a computer software program, Research Randomizer. The group assignments were placed in sealed envelopes and opened sequentially at the time of randomization.

### Sample size

Sample size was calculated using the GPower 3.1.9.7 software. The software supports a priori sample size calculation by imputing the effect size, desired alpha level, and power level (23). The sample size required for an effect size of 0.2 from a previous similar study (22) and for a margin of error of 0.05 was estimated as being a minimum of 60 participants for each group, assuming a power of 80%. Considering 20% as the likely drop-out rate during the study, the total sample size needed was 72 participants per group, i.e., a total of 216 participants.

### Interventions

This was a three-month program. There were three groups in the study: the mHealth group, the mHealth with interactivity group (mHealth+I), and the control group. Details of the intervention are found in our published protocol (24).

### mHealth group

Trained staff from the community center helped the participants in the mHealth group download and use an mHealth app developed by the research team and a telecommunication company. The app has several features, including the monitoring of vital signs, scheduling of appointments, notification of medications, and the dissemination of updated health education. A nurse monitored the vital signs of the participants daily in the app database and when abnormalities were found, she would call and assess the participants within 24 h *via* smartphone and follow the working protocol to either educate the participants in self-care techniques and knowledge or refer the participants to a hospital. The working protocol was developed based on the Omaha System (25) and guidelines from the National Institute for Health and Care Excellence (26). There was also one button that was installed in the app for participants to call the nurse when they considered it to be necessary. The main purpose of the app was to empower the participants to self-manage the three common problems (i.e., pain, hypertension, and diabetes) encountered in late life. The participants were told to use the app daily during the 3-month intervention period. When they had not used it for more than 1 week, a reminder message would pop up on the smartphone screen.

### mHealth with interactivity group (mHealth+I)

In addition to the use of the mHealth app, participants in the mHealth+I group received eight proactive calls from a nurse over the 3-month program period (i.e., First month: weekly calls; Second and third months: biweekly calls). In these eight telephone calls, the nurse not only assessed the physical health of the participants, but also their psychological health by using a holistic assessment tool, the Omaha System. The Omaha system was adopted to identify the needs and chief complaints of an individual in four domains: environmental, psychosocial, physiological, and health-related behavior (25). It was found to be applicable for older adults in the community and proven valid for local use (20). Following an assessment, the nurse engaged and empowered the participants to set self-help goals and realistic action plans, and provided psychological support by giving positive verbal encouragement during the conversation. When deemed necessary, the nurse would refer the participants to our health-social partnership team, which included social workers and a general practitioner, according to a team-decided referral protocol. The social workers provided home and meal delivery services, counseling, and financial support, while the general practitioner provided medical consultations, and treatment and procedures to the participants. A biweekly case conference was held between the nurse, the social workers, and the general practitioners to discuss the progress and updated conditions of the subjects, suggest revising or modifying the contract goals, and address the concerns of the subjects.

### Control group

The participants in the control group did not receive the mHealth app or proactive calls from nurses.

### Data collection

Data were collected at three time-points: pre-intervention (T1), 3 months post-intervention (T2), and 3 months after the completion of the program (T3) to measure the sustained effect of the program. The research assistants, who were trained and blinded to the group assignments, were responsible for collecting the data in the five community centers.

### Outcome measures

Quality of life was measured using the 12-item Short Form Health Survey version 2—Chinese (HK) version (SF12v2) (27). The 12 items in the questionnaire were rated on Likert-type scales and summed to provide easily interpretable scales for physical component summary (PCS) and mental component summary (MCS). PCS includes physical functioning, bodily pain, and role-physical, whereas MCS encompasses social functioning, role-emotional, and mental health (28). Higher scores in both components indicated better quality of life. The validity and reliability of the scale have been confirmed in numerous studies (29–31). Higher scores indicate a better quality of life.

Self-efficacy was measured using the General Self-efficacy scale (GCSE) (32). The scores for this 10-item Likert scale ranged from 10 to 40, with higher scores indicating better self-efficacy. The scale showed excellent reliability for Chinese older adults (32).

Depression was assessed using the Geriatric Depression Scale (GDS). This 15-item questionnaire was used to explore the participants' feelings with dichotomous answers. Scores from each item were added for a total possible score of 15, with higher scores representing a higher severity of depressive symptoms. Good reliability, validity, and factor structure were demonstrated (33).

Demographic data were collected at baseline, including information on gender, age, marital status, living conditions, caregiver, and frequency of care.

### Data analysis

The data were analyzed using the Statistical Package for the Social Sciences (SPSS) version 26 software. Baseline demographic data were presented using count numbers and percentages. The outcomes for each group were presented in terms of mean, standard deviation, and 25th and 75th percentiles. The Generalized Estimating Equation was adopted to evaluate the between-group, within-group, and interaction effects between time and group for each outcome since it does not require the outcome variable to have a normal distribution (34). This feature could be highly beneficial to this study as we anticipated that outcomes such as self-efficacy and depression for community-dwelling older adults would be heavily skewed. Intention-to-treat was used as the primary analysis in this study. *P* < 0.05 were regarded as significant for a two-tailed test.

### Ethical considerations

Ethical approval for the study was obtained from the Ethics Sub-committee of the Hong Kong Polytechnic University (HSEARS20190312002). Written consent was received from all participants. Participants were assured that they could withdraw from the study at any time without any adverse consequences. The data collected were encrypted in a password-protected database.

## Results

### Participant flow

**Figure 1:**
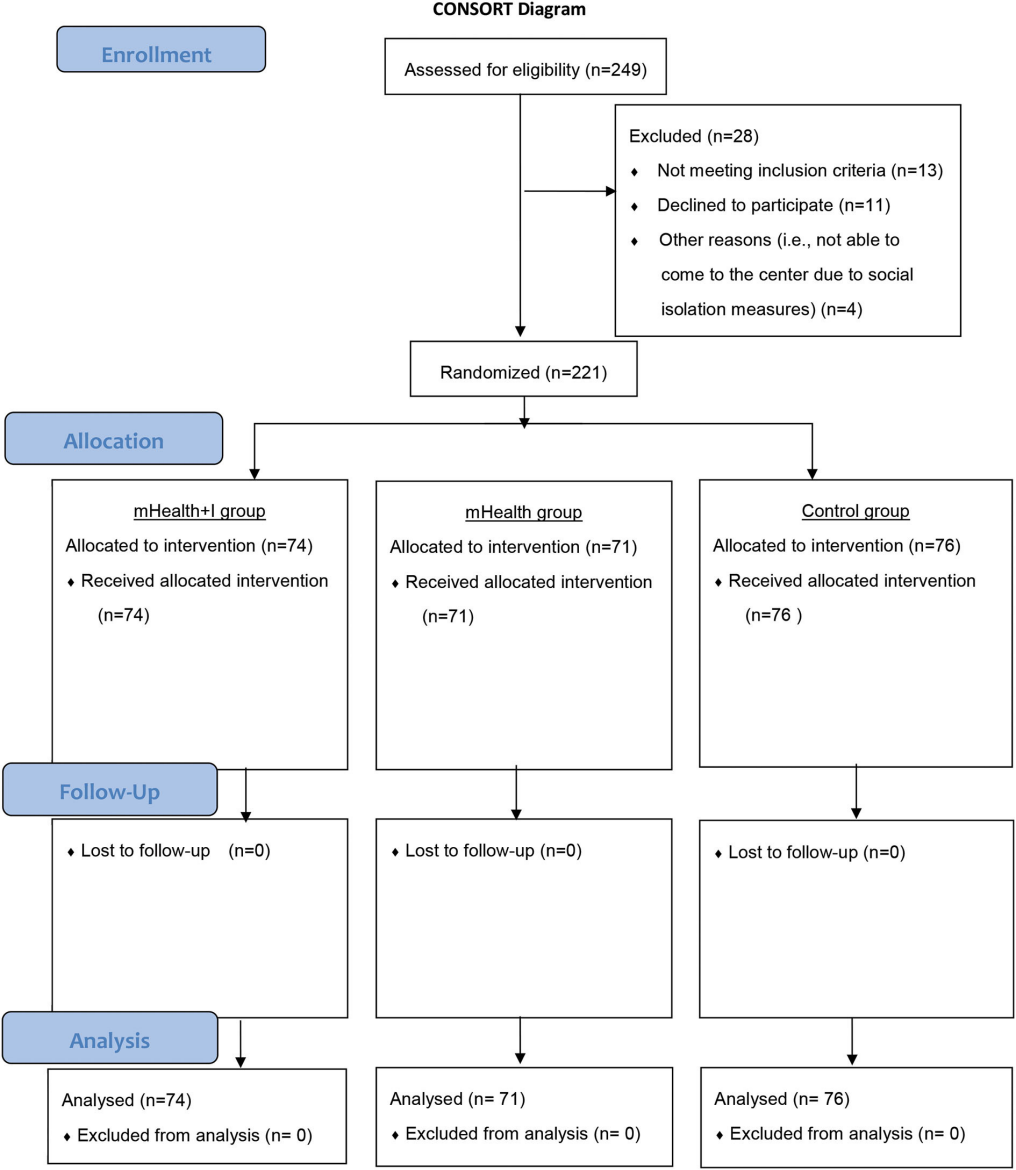
CONSORT table.

Of the 249 potential community-dwelling older adults who were assessed for eligibility, 221 agreed to join the program and were randomized into the mHealth+I (*n* = 74), mHealth (*n* = 71), or control groups (*n* = 76). No participant dropped out from the program. Figure 1 shows the CONSORT diagram.

### Sample description

There were no statistically significant differences in demographic characteristics among the three groups at baseline. Regarding marital status, most participants were married (45.7%) or widowed (47.1%). The majority lived in a flat (98.2%) with either family members (43.0%) or their spouse (26.2%), while 30.8% lived alone. Many reported that they took care of themselves (70.1%). Some were taken care of by their children (41.6%), their spouse (24.4%), or siblings (10.4%). More than half claimed that they always received care from others when needed (65.6%). The baseline demographic characteristics of each group are reported in Table 1.

**Table 1:** Demographic characteristics of the participants.

		**Total**		**mHealth** + **I group**		**mHealth group**		**Control group**		***p-value*** ***Chi-square test/ Kruskal-Wallis Test***
		**(*****n*** = **221)**		**(*****n*** = **74)**		**(*****n*** = **71)**		**(*****n*** = **76)**		
		**Count**	**Table** **valid** ***N*** **%**	**Count**	**Column** **valid** ***N*** **%**	**Count**	**Column** **valid** ***N*** **%**	**Count**	**Column** **valid** ***N*** **%**	
Gender	Male	36	16.3%	14	18.9%	9	12.7%	13	17.1%	0.579
	Female	185	83.7%	60	81.1%	62	87.3%	63	82.9%	
Age	Mean (SD)	76.56	(7.96)	74.69	(7.57)	77.63	(7.84)	77.38	(8.21)	0.083
	Median (range)	76	(60–98)	73.50	(60–98)	78.0	(60–91)	77.50	(63–95)	
Marital status	Single	5	2.30%	2	2.70%	1	1.40%	2	2.60%	0.308
	Married	101	45.7%	39	52.7%	24	33.8%	38	50.0%	
	Divorced	11	5.00%	3	4.10%	5	7.00%	3	3.90%	
	Widowed	104	47.1%	30	40.5%	41	57.7%	33	43.4%	
Living conditions	Flat	217	98.2%	72	97.3%	70	98.6%	75	98.7%	0.735
	Subdivided flat	2	0.90%	1	1.40%	1	1.40%	43	0.00%	
	Cage home	2	0.90%	1	1.40%	0	0.00%	71	1.30%	
	Others	0	0.00%	0	0.00%	0	0.00%	5	0.00%	
Living	Alone	68	30.8%	21	28.4%	31	43.7%	16	21.1%	0.065
	With spouse	58	26.2%	19	25.7%	13	18.3%	26	34.2%	
	With family	95	43.0%	34	45.9%	27	38.0%	34	44.7%	
Caregiver: self	Yes	155	70.1%	55	74.3%	47	66.2%	53	69.7%	0.562
	No	66	29.9%	19	25.7%	24	33.8%	23	30.3%	
Caregiver: spouse	Yes	54	24.4%	18	24.3%	16	22.5%	20	26.3%	0.867
	No	167	75.6%	56	75.7%	55	77.5%	56	73.7%	
Caregiver:	Yes	23	10.4%	7	9.50%	8	11.3%	8	10.5%	0.938
siblings	No	198	89.6%	67	90.5%	63	88.7%	68	89.5%	
Caregiver:	Yes	92	41.6%	34	45.9%	27	38.0%	31	40.8%	0.616
children	No	129	58.4%	40	54.1%	44	62.0%	45	59.2%	
Caregiver:	Yes	5	2.30%	2	2.70%	1	1.40%	2	2.60%	0.841
Children-in-law	No	216	97.7%	72	97.3%	70	98.6%	74	97.4%	
Caregiver:	Yes	3	1.40%	1	1.40%	1	1.40%	1	1.30%	0.999
Friends	No	218	98.6%	73	98.6%	70	98.6%	75	98.7%	
Caregiver:	Yes	7	3.20%	1	1.40%	3	4.20%	3	3.90%	0.547
Neighbors	No	214	96.8%	73	98.6%	68	95.8%	73	96.1%	
Caregiver:	Yes	8	3.60%	4	5.40%	2	2.80%	2	2.60%	0.600
Volunteers	No	213	96.4%	70	94.6%	69	97.2%	74	97.4%	
Caregiver:	Yes	19	8.60%	7	9.50%	5	7.00%	7	9.20%	0.850
Domestic helpers	No	202	91.4%	67	90.5%	66	93.0%	69	90.8%	
Frequency of care	Always	145	65.6%	49	66.2%	41	57.7%	55	72.4%	0.510
	Sometimes	37	16.7%	14	18.9%	13	18.3%	10	13.2%	
	Only at night	11	5.00%	2	2.70%	6	8.50%	3	3.90%	
	No help from others	28	12.7%	9	12.2%	11	15.5%	8	10.5%	

SD, standard deviation.

### Effectiveness of the interventions on outcomes

#### Quality of life

**Figure 2:**
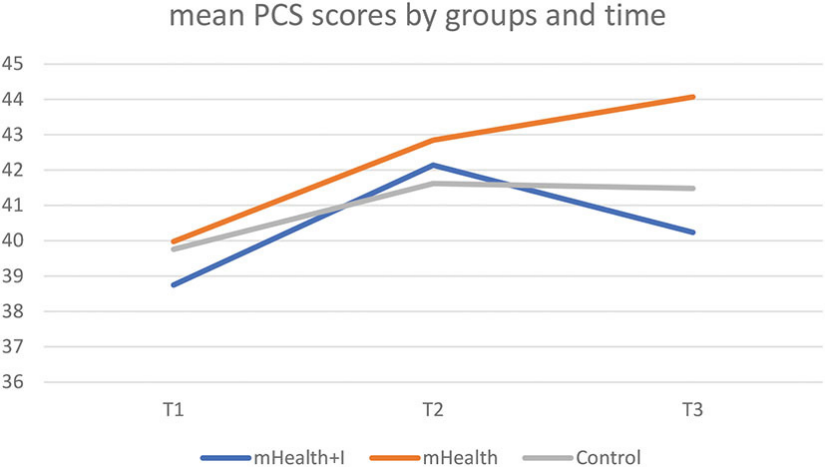
Mean PCS scores by three groups and time.

**Figure 3:**
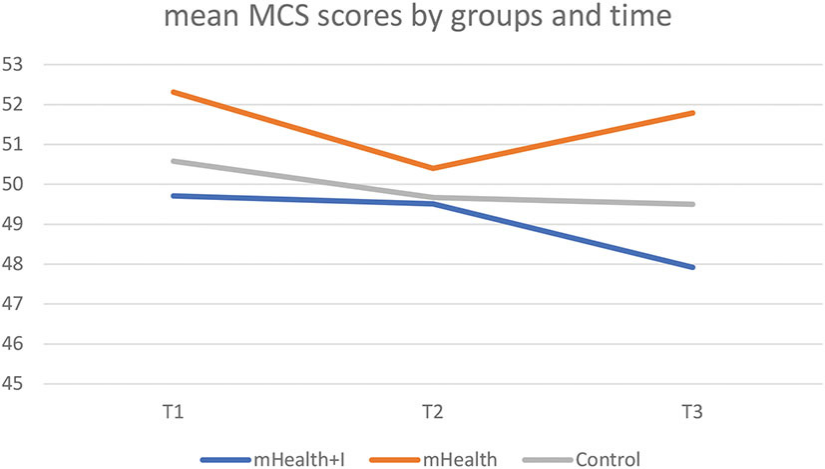
Mean MCS scores by three groups and time.

The Physical Component Summary (PCS) and Mental Component Summary (MCS) scores were derived from the SF-12v2. As seen in **Table 3**, there were no statistically significant between-group, within-group, and interaction effects between group and time in both the PCS and MCS scores in the three groups. Only the mHealth group exhibited an improvement in mean PCS scores from T1 to T3 (Figures 2, 3).

#### Self-efficacy

**Figure 4:**
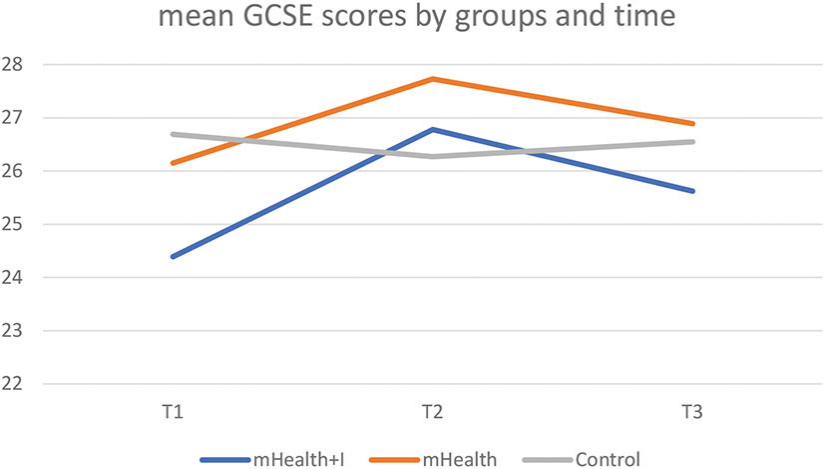
Mean GCSE scores by three groups and time.

**Table 3** shows that there was statistically significant improvement in GCSE scores within the mHealth+I group [β = −2.31 (95%CI of β, −4.26 to −0.36), *p* = 0.020]. The GEE analysis revealed that there was also an interaction effect between the mHealth+I group in T2 and the control group in T1 [β = 2.81 (95%CI of β, 0.74–4.89), *p* = 0.008]. However, Table 2 and Figure 4 showed that the GCSE scores of the mHealth+I group dropped from T2 to T3 after an improvement from T1 to T2, which indicated that the intervention effect was not maintained 3 months after the completion of the program.

**Table 2:** The mean differences of the outcomes between T1 and T2 for each group.

**Outcomes**	**Groups**		**Mean**	**Standard deviation**	**Percentile**	
					**25**	**75**
Quality of life—PCS	Control group	T3	41.48	9.32	35.66	48.81
		T2	41.62	8.17	35.14	48.35
		T1	39.76	10.27	32.99	47.84
	mHealth group	T3	44.07	8.52	38.72	49.67
		T2	42.85	10.57	36.31	49.87
		T1	39.98	10.20	33.12	48.35
	mHealth+I group	T3	40.24	9.82	31.82	47.76
		T2	42.14	8.99	34.66	50.01
		T1	38.75	9.33	33.36	44.52
Quality of life—MCS	Control group	T3	49.50	12.67	42.68	59.64
		T2	49.67	11.01	44.60	57.30
		T1	50.58	11.56	44.40	59.52
	mHealth group	T3	51.79	10.69	44.00	59.94
		T2	50.40	10.15	44.00	59.30
		T1	52.31	10.94	44.50	60.53
	mHealth+I group	T3	47.92	10.39	40.13	52.97
		T2	49.51	9.43	45	56.29
		T1	49.71	10.80	42.18	59.19
Self-efficacy	Control group	T3	26.55	6.53	24.68	30.24
		T2	26.28	5.33	23.50	29.50
		T1	26.70	6.36	22.50	30.00
	mHealth group	T3	26.89	5.84	24.00	30.00
		T2	27.73	5.22	24.00	30.00
		T1	26.15	6.88	22.00	31.00
	mHealth+I group	T3	25.62	7.13	21.00	30.00
		T2	26.78	6.39	22.00	30.00
		T1	24.39	5.90	21.00	29.00
Depression	Control group	T3	3.92	3.38	1.05	6.02
		T2	3.75	3.08	1.00	6.00
		T1	3.63	3.26	1.00	6.00
	mHealth group	T3	3.24	3.33	1.05	6.05
		T2	3.54	3.14	1.00	5.00
		T1	4.08	3.77	1.00	7.00
	mHealth+I group	T3	4.41	3.46	2.05	6.11
		T2	3.81	2.84	2.00	5.00
		T1	4.47	3.59	2.00	7.00

PCS, physical component summary; MCS, mental component summary.

#### Depression

**Figure 5:**
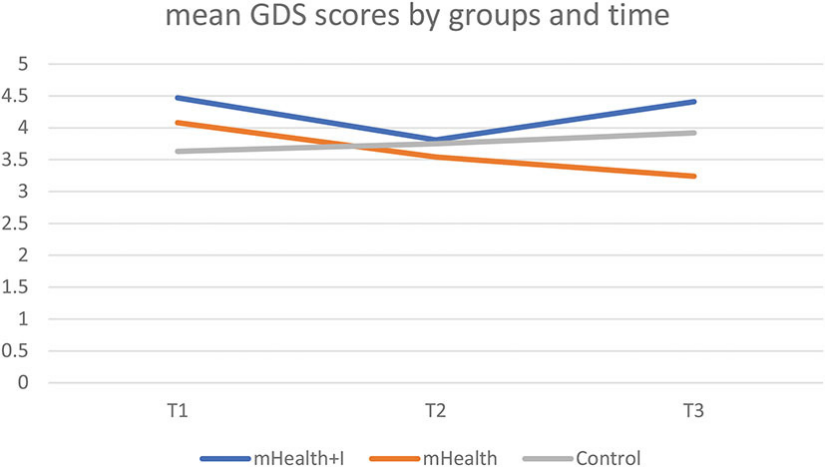
Mean GDS scores by three groups and time.

The baseline mean scores for all three groups were below the cut-off point of 5 (35). Table 2 showed that the mHealth group experienced an improvement in mean GDS scores from T1 to T3. There was a statistically significant interaction effect between the mHealth group in T3 and the control group in T1 [β = −1.14 (95%CI of β, −2.20 to −0.07), *p* = 0.04] (Table 3). Figure 5 illustrates the mean GDS scores by group and time.

**Table 3:** Parameter estimates of outcomes.

	**B**	**SE**	**95% CI**		**Wald χ^2^**	**Sig**.
			**Lower**	**Upper**		
**Quality of life—PCS**						
(Intercept)	39.76	1.17	37.47	42.06	1,153.62	< 0.001*
mHealth+I group	−1.01	1.59	−4.13	2.11	0.40	0.53
mHealth group	0.22	1.68	−3.07	3.50	0.02	0.90
Time = 3	1.72	1.28	−0.78	4.23	1.82	0.18
Time = 2	1.86	0.99	−0.08	3.80	3.52	0.06
mHealth + I group* Time = 3	−0.23	1.78	−3.73	3.26	0.02	0.90
mHealth + I group * Time = 2	1.53	1.52	−1.45	4.52	1.01	0.31
mHealth group* Time = 3	2.37	1.80	−1.16	5.89	1.73	0.19
mHealth group * Time = 2	1.02	1.76	−2.44	4.48	0.33	0.56
**Quality of life—MCS**						
(Intercept)	50.58	1.32	48.00	53.16	1,473.94	< 0.001*
mHealth+I group	−0.87	1.81	−4.42	2.69	0.23	0.63
mHealth group	1.73	1.84	−1.89	5.34	0.88	0.35
Time = 3	−1.08	1.47	−3.96	1.80	0.55	0.46
Time = 2	−0.91	1.03	−2.92	1.10	0.79	0.37
mHealth+I group* Time = 3	−0.71	2.00	−4.63	3.20	0.13	0.72
mHealth+I group * Time = 2	0.71	1.63	−2.48	3.90	0.19	0.66
mHealth group* Time = 3	0.57	2.06	−3.47	4.60	0.08	0.78
mHealth group * Time = 2	−0.99	1.62	−4.17	2.19	0.37	0.54
**Self-efficacy**						
(Intercept)	26.70	0.73	25.28	28.12	1,355.10	< 0.001*
mHealth + I group	−2.31	0.99	−4.26	-0.36	5.37	0.02
mHealth group	−0.54	1.09	−2.68	1.59	0.25	0.62
Time = 3	−0.15	0.78	−1.67	1.38	0.03	0.85
Time = 2	−0.42	0.81	−2.02	1.17	0.27	0.61
mHealth + I group* Time = 3	1.37	1.02	−0.63	3.38	1.81	0.18
mHealth+I group * Time = 2	2.81	1.06	0.74	4.89	7.07	0.008
mHealth group* Time = 3	0.88	1.07	−1.22	2.98	0.67	0.41
mHealth group * Time = 2	2.00	1.09	−0.14	4.13	3.37	0.07
**Depression**						
(Intercept)	3.63	0.72	2.90	4.36	95.73	< 0.001*
mHealth + I group	0.84	0.56	−0.25	1.93	2.29	0.13
mHealth group	0.45	0.58	−0.68	1.59	0.61	0.43
Time = 3	0.29	0.40	−0.49	1.07	0.53	0.47
Time = 2	0.12	0.35	−0.57	0.81	0.11	0.74
mHealth + I group* Time = 3	−0.36	0.52	−1.38	0.67	0.47	0.49
mHealth + I group * Time = 2	−0.78	0.47	−1.71	0.15	2.72	0.10
mHealth group* Time = 3	−1.14	0.54	−2.20	-0.07	4.39	0.04
mHealth group * Time = 2	−0.67	0.54	−1.72	0.38	1.56	0.21

SE, standard error; *p < 0.05.

## Discussion

The increasing emergence of mHealth applications warrants an evaluation of their impact not only on physical health, but also on psychological outcomes. The current study examined the added benefit of including nurse-directed telephone follow-up calls supported by a health-social partnership approach in the use of mHealth, as well as the sustained effects on psychological outcomes among community-dwelling older adults. The findings of the study highlight the point that whereas the mHealth group showed improved PCS and depression scores from T1 to T2, which were sustained at T3, the mHealth+I group demonstrated improved self-efficacy from T1 to T2 with a decrease at T3. Overall, the study's findings may offer evidence regarding the positive impact of the mHealth app on some psychological outcomes. However, adding the nurse-directed telephone calls may be of little to no benefit in the long term.

The sustained beneficial effects on PCS and depression levels in the mHealth group participants indicated that the impact of the mHealth application lasted beyond the immediate post-intervention phase. The mHealth application employed in this study offered a one-stop shop for community-dwelling older adults to monitor their vital parameters and facilitated exchanges with a healthcare provider. These active, ongoing interactive processes may have helped to ease the distress associated with not knowing what one's symptoms meant and offering access to professional support if required [Hernandez (36)]. Besides, the interactive platform offered a convenient mode of delivery for health interventions and facilitating self-management, which may have put the minds of these older adults at ease to actively participate in their care during the intervention period (37).

The study also observed improved self-efficacy within the mHealth+I group from T1 to T2. However, the self-efficacy scores dropped at T3, demonstrating that the improvement in self-efficacy that was observed could not be sustained after the completion of the program. Self-efficacy refers to the perceived capability or belief that one can perform a targeted behavior (38). It remains a robust predictor of various health behaviors (39). An increase in the level of self-efficacy is often associated with the perception of a lowering of barriers to engaging in health behaviors, which in turn translates into an increased level of motivation to participate in the required activity (40). It is possible that the gradual increase in the self-efficacy of the older adults as observed at T2 was due to the consistent engagement and support from the health-social partnership team during the intervention period. When the support ended at T2, their levels of self-efficacy dropped. This finding may imply that a longer period may be required to build and sustain self-efficacy levels among community-dwelling older adults. This is particularly important as community-dwelling older adults are a heterogeneous group with diverse chronic illnesses. Considering that the impact of these chronic illnesses may differ, different durations of support may be required for different individuals if self-efficacy levels are to be sustained. In future studies aimed at improving and sustaining improved levels of self-efficacy over longer periods, it may therefore be necessary to consider this factor in the design and implementation of interventions. Also, a gradual approach to weaning older adults off formal support should be considered if such support is included in an intervention.

## Related works

Although no study was identified regarding the sustained effects of mHealth applications on psychological outcomes for older adults, some studies have reported positive sustained effects following the utilization of mHealth apps. For instance, in a recent systematic review and meta-analysis of seven studies, the authors observed that the mHealth app had a sustained positive effect on anxiety and depression during the follow-up at 11 weeks (41). In another systematic review and meta-analysis of 28 studies, it was observed that mHealth interventions improved the mental health outcomes of employees and that the effects were sustained across varying time-points (42). A possible reason for this result could be that frequent use of the mHealth app over the study period may have helped the participants to acquire and apply self-care skills and knowledge and to note the positive changes in their health, which might have given them the motivation to continue using the app after the program. There was also another systematic review of eight studies showing that the benefits of app usage such as reducing depression, stress, and substance use among people with all ages can be maximized with the support of mental health professionals (37). However, the small number of studies and participants included in each of the studies, the high risk of bias, and unknown efficacy of long-term follow-up warrant the need for more scientific evidence.

Similar to the finding observed regarding improved self-efficacy in the mHealth+I group, other studies have also reported improved self-efficacy among community-dwelling older adults. For instance, Müller et al. (43) reported an improvement in self-efficacy following the implementation of a mHealth app which comprised of SMS text-messaging and follow-up reminders for older adults over a 12-week period. Physical activity self-efficacy was also reported to have improved among older adults following the implementation of a physical activity mHealth app (44). Despite the improvement in self-efficacy, Fanning et al. (44) did not observe any meaningful improvement in the quality of life of the older adults despite the comprehensive mHealth app that was implemented. Conversely, Christiansen et al. (45) observed improved quality of life following the implementation of a mHealth app for older adults with mild cognitive impairment noting that having moderately or high technical skills in using mHealth technology and using the internet *via* mHealth technology on a daily or weekly basis was associated with good to excellent QoL. Despite the mixed findings, these studies did not examine the sustained effects of the mHealth apps beyond the intervention duration which makes it rather difficult to draw stronger conclusions in relation to the current study. While more work may be needed to understand this phenomenon, the emerging evidence seems to suggest that mHealth applications may have a positive impact on psychological outcomes in both the short and medium term.

## Limitations

Some limitations were noted in this study. First, the study was not able to capture the long-term effect of the intervention (i.e., longer than 6 months) due to the limited time and budget involved. Second, the results are not generalizable to older adults who have no smartphone or Internet coverage at home. Third, the nurse in the study did not provide immediate help because she worked only during office hours. Fourth, the study did not collect objective data. The subjective data collected *via* self-reported questionnaires in this study may create bias to the result. Regardless of these limitations, the findings of the present study are likely to have a meaningful impact on bringing psychological relief to community-dwelling older adults.

## Conclusion

The results of the present study suggest that the therapeutic effects of an mHealth app on community-dwelling older adults can be improved or maintained with regard to psychological outcomes, which include quality of life, self-efficacy, and depression. Future research should focus on ways to motivate older adults to use the mHealth app, in order to maximize its benefits.

## Data availability statement

The raw data supporting the conclusions of this article will be made available by the authors, without undue reservation.

## Ethics statement

The studies involving human participants were reviewed and approved by the Research Committee of the Hong Kong Polytechnic University. The participants provided their written informed consent to participate in this study.

## Author contributions

AW and FW designed the study. AW, JB, and AL drafted the article, which was critically revised by all of the authors. KC and SW coordinated the data collection and supervised the quality of the program. AW conducted the statistical analysis. AW, FW, JB, KC, SW, and AL had full access to interim reports on statistics, analyses, and tables. All authors contributed to the article and approved the submitted version.

## Funding

This work was supported by a grant from the Nethersole Institute of Continuing Holistic Health Education (NICHE) (Ref No. ZH4B). The funding organization had no role in the study design, data collection, data analysis, data interpretation, writing of the report, or the decision to publish the study.

## Conflict of interest

The authors declare that the research was conducted in the absence of any commercial or financial relationships that could be construed as a potential conflict of interest.

## Publisher's note

All claims expressed in this article are solely those of the authors and do not necessarily represent those of their affiliated organizations, or those of the publisher, the editors and the reviewers. Any product that may be evaluated in this article, or claim that may be made by its manufacturer, is not guaranteed or endorsed by the publisher.
